# Performance Characteristics of a PEM Fuel Cell with Parallel Flow Channels at Different Cathode Relative Humidity Levels

**DOI:** 10.3390/s91109104

**Published:** 2009-11-17

**Authors:** Pil Hyong Lee, Sang Soon Hwang

**Affiliations:** Department of Mechanical Engineering, University of Incheon, Songdo Dong, Yeonsu-Ku, Incheon, Korea; E-Mail: meman80@incehon.ac.kr

**Keywords:** humidity, micro parallel flow channels, fuel cell, numerical simulation

## Abstract

In fuel cells flow configuration and operating conditions such as cell temperature, humidity at each electrode and stoichiometric number are very crucial for improving performance. Too many flow channels could enhance the performance but result in high parasite loss. Therefore a trade-off between pressure drop and efficiency of a fuel cell should be considered for optimum design. This work focused on numerical simulation of the effects of operating conditions, especially cathode humidity, with simple micro parallel flow channels. It is known that the humidity at the cathode flow channel becomes very important for enhancing the ion conductivity of polymer membrane because fully humidified condition was normally set at anode. To investigate the effect of humidity on the performance of a fuel cell, in this study humidification was set to 100% at the anode flow channel and was changed by 0–100% at the cathode flow channel. Results showed that the maximum power density could be obtained under 60% humidified condition at the cathode where oxygen concentration was moderately high while maintaining high ion conductivity at a membrane.

## 1. Introduction

**Figure 1.**
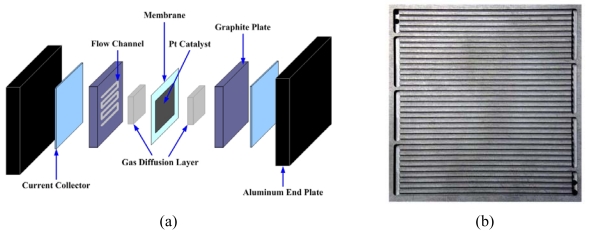
Schematics of a fuel cell assembly displaying different essential components of the system (a) and micro serpentine bipolar flow plate (b).

The PEM (Proton Exchange Membrane) Fuel Cell is an electrochemical device that directly converts chemical energy into electrical energy. Its features, such as high power density, simple construction, and fast startup make it suitable for applications in automotive and domestic appliances [1-3]. A typical schematic of a Proton Exchange Membrane Fuel Cell (PEMFC) is shown in Figure 1(a) The cell is a sandwich of two graphite bipolar plates with micro flow channels and separated by MEA (Membrane Electrode Assembly) which consists of a membrane and two electrode with dispersed Pt catalyst. The gas diffusion layer (GDL) is porous to supply reactants to the electrodes in the unexposed areas of the micro flow channels [4].

As shown Figure 1(b), typical serpentine bipolar flow plates in fuel cells contain many micro flow channels to distribute the reactant gas flow over the catalytic reactive surface. The shape, size and pattern of micro flow channels are known to greatly affect fuel cell performance.

Selection of operating conditions for PEMFC with parallel, serpentine, interdigitated flow channels are very important to improve the performance of fuel cells. Generally the operating pressure and temperature, equivalence ratio are known to be key elements to increase its performance and their effects on performance were studied by many researchers. However little study on the effects of humidity in air and hydrogen has been carried out until now. Wang *et al.* [5] reported that the reactant relative humidity and the flow field design significantly affect cell performance. For the same operating conditions and reactant relative humidity, the interdigitated design has better cell performance than the parallel design. With a constant anode relative humidity = 100%, for lower operating voltages, a lower cathode relative humidity reduces cathode flooding and improves cell performance, while for higher operating voltages, a higher cathode relative humidity maintains the membrane hydration to give better cell performance. Ohsaka *et al.* [6] showed that symmetric relative humidity has different impact, depending on the cell temperature. While at relative humidity of 35% the cell showed considerable performance at a cell temperature = 70 °C, it was not so at a cell temperature of 90 °C. At cell temperature of 70 °C, the cell potential increases with relative humidity at lower and medium current densities, but decreases with relative humidity at higher currents. In this study, we attempt to develop a fully three dimensional computational model for PEM fuel cell which can deal with both anode and cathode micro parallel flow channels. To investigate the effect of humidity on performance of fuel cell, humidification condition is set to 100% at the anode flow channel and is changed by 0–100% at the cathode flow channel. The commercial program FLUENT (Version 6.3) was modified using UDF (User-Defined Functions) in order to simulate electrochemical reactionz and related phenomena that occurred in a PEMFC with micro parallel flow channel. [7-13] The distribution of water and oxygen concentration along the micro parallel flow channel and gas diffusion layer, water transport though membrane, and membrane ion conductivity were investigated and analyzed.

## 2. Numerical Models

### 2.1. Model Assumptions

Governing equations for calculating the fully three dimensional flow channel are expressed under following assumptions:

1. The gas mixture is incompressible, ideal fluid;
2. The flow in the flow channel is laminar (Reynolds number <900 at anode and cathode relative humidity 100%);
3. Isothermal condition;
4. Butler-Volmer kinetics for electrochemical reaction rate.

### 2.2. Governing Equations

1. Mass conservation equation:
(1) $$\nabla \cdot (ɛ\rho \vec{u})=S_{m}$$
  where *ε* is the porosity of the porous media, which is equal to unit for the gas channels, *ρ* the density, and *ū* the intrinsic fluid velocity vector, *εū*, reflects the superficial velocity in the porous media. *S_m_* denotes source terms corresponding to the consumption of hydrogen and oxygen in the anode and cathode, and the production of water in the cathode:
(2) $$\begin{array}{l} S_{m}=S_{H_{2}}+S_{aw}:\text{Anode Side}\\ S_{m}=S_{O_{2}}+S_{aw}:\text{Cathode Side} \end{array}$$
2. Momentum conservation equation
  The fluid flow in the fuel cell can be described by the general equation as:
(3) $$\nabla (ɛ\rho \vec{u}\vec{u})=-ɛ\nabla p+\nabla (ɛ\mu \nabla \vec{u})+S_{u}$$
  where *ρ* denotes the pressure, *μ* the effective viscous coefficient. Because the fluid flowing in the channels, gas diffusion layers and catalyst layer membrane is different, *μ* stand, for gas viscous coefficient for gas mixture in the channel and gas diffusion layer, and liquid viscous coefficient for liquid in the catalyst layer and membrane. Furthermore, mass-weighted mixing law gives viscosity of the gaseous mixture. The source terms in the momentum equations are added based on the Darcy's law, representing an extra drag force in the equation as follows:
(4) $$S_{ux}=-\frac{\mu u}{\beta_{x}},S_{uy}=-\frac{\mu v}{\beta_{y}},S_{uz}=-\frac{\mu w}{\beta_{z}}$$
  where *β_x_*, *β_y_* and *β_z_* are the permeability in the x, y, z direction and u, v and w are the velocities in x, y and z directions respectively.
3. Species conservation equation
  The species conservation equation for the gas mixture is:
(5) $$\nabla (ɛ\vec{u}C_{k})=\nabla (D_{k}^{eff}\nabla C_{k})+S_{k}$$
  Here, *k* denotes chemical species that include hydrogen, oxygen, nitrogen and water. $D_{k}^{eff}$is the effective diffusion coefficient. Source term *S_k_* denotes:
(6) $$S_{k}=\left\{ \begin{array}{l} -\frac{I(x,y)}{2F}M_{H_{2}}A_{cv}:S_{H_{2}}\\ -\frac{\alpha (x,y)}{F}I(x,y)M_{H_{2}O}A_{cv}:S_{aW}\\ -\frac{I(x,y)}{4F}=M_{O_{2}}A_{cv}:S_{O_{2}}\\ \frac{1+2\alpha (x,y)}{2F}I(x,y)M_{H_{2}O}A_{cv}:S_{cW} \end{array}\right.$$
  where *M*_*H*_2__, *M*_*H*_2_*O*_ and *M*_*O*_2__ are the molecular weight of hydrogen, water and oxygen.

### 2.3. Water Transport Equation

Water management is a critical issue for the performance of a proton electrolyte membrane fuel cell. The transport phenomena of water can be described as follows: the water molecules are transported through the polymer electrolyte membrane by the protons and this process is called electro-osmotic drag. In addition to the molecular diffusion and electro-osmotic drag, water is generated in the cathode catalyst layer due to electro chemical reaction.

1. Electro-osmotic drag flux
  Electro-osmotic water flux through the membrane can be calculated from the proton flux through the membrane, given by the specified current density and Faraday's law:
(7) $$J_{H_{2}O}=2\times n_{d}\frac{I(x,y)}{2F}:\text{Electro}–\text{osmotic drag flux}$$
  where *n_d_* is Electro-osmotic drag coefficient which depends on water activity as follows:
(8) $$n_{d}=0.0029\lambda^{2}+0.05\lambda -3.4\times 10^{-19}$$
  where *λ* represents water contend of the membrane described as:
(9) $$\begin{array}{l} \lambda =0.043+17.81a_{K}-39.85a_{K}^{2}+36.0a_{K}^{3},0<a_{K}<1;\\ \lambda =14+1.4(a_{K}-1),1<a_{K}\leq 3 \end{array}$$
  where *a_K_*, water activity, is expressed as:
(10) $$a_{K}=\frac{X_{w,K}P(x,y)}{P_{w,K}^{\text{sat}}},K=\text{Anode or Cathode}$$
  where *X_w,K_*, *P^sat^* are water mole fraction and saturation pressure at each electrode respectively:
(11) $${\text{log}}_{10}P^{\text{sat}}=-2.1794+0.02953T-9.1837\times 10^{-5}T^{2}+1.4454\times 10^{-7}T^{3}$$
2. Back diffusion flux
  The water formation at the cathode results in a gradient in the water content between the cathode side and anode side of the membrane. For PEMFC, this gradient causes a water flux back to the anode side which is superimposed to the electro-osmotic flux. This back diffusion is expressed as following water flux:
(12) $$J_{H_{2}O,\text{back diffusion}}=-\frac{\rho_{m,\text{dry}}}{M_{m,\text{dry}}}\times D_{w}\times \frac{d\lambda }{dy}:\text{Back diffusion flux}$$
  where *ρ_m,dry_* is the dry density of electrolyte, *M_m,dry_* is the electrolyte equivalent weight, and *z* is the direction through the membrane thickness.
  *D_w_* is water diffusion coefficient which is strongly dependent on water content as follows:
(13) $$\begin{array}{l} D_{w}=D_{\lambda }\text{exp}\left(2416\left(\frac{1}{303}-\frac{1}{T_{\text{cell}}}\right)\right);D_{\lambda }=10^{-10},\lambda <2\\ D_{\lambda }=10^{-10}(1+2(\lambda -2)),2\leq \lambda \leq 3;\\ D_{\lambda }=10^{-10}(3-1.67(\lambda -3)),3<\lambda <4.5;\\ D_{\lambda }=1.25\times 10^{10},\lambda \geq 4.5 \end{array}$$
3. Current density and membrane ion Conductivity
  *I*(x,y,z) is current density generated by electrochemical reaction, which can be expressed as:
(14) $$I(x,y,z)=\frac{\sigma_{m}(x,y,z)}{t_{m}}\{V_{oc}-V_{\text{cell}}-\eta (x,y,z)\}$$
  where *σ_m_*(x,y,z) indicates the ion conductivity of membrane expressed as:
(15) $$\sigma_{m}(x,y,z)=\left(0.00514\frac{M_{m,\text{dry}}}{\rho_{m,\text{dry}}}C_{w,a}(x,y,z)-0.00326\right)\text{exp}\left(1268\left(\frac{1}{303}-\frac{1}{T_{s}}\right)\right)\times 10^{2}$$

### 2.4. Numerical Simulation Model

**Figure 2.**
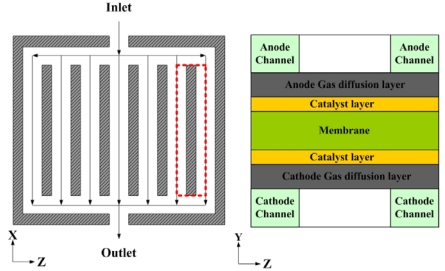
Schematic of the micro parallel flow channel structure.

A schematic of the micro parallel flow channel with GDL and catalyst layer is shown in Figure 2. Cross sectional area at inlet of micro parallel flow channel is 762 × 762 (μm) and its length is 40 (mm). Thickness of the gas diffusion layer and catalyst layer is set to 254 (μm) and 28.7 (μm), respectively.

**Figure 3.**
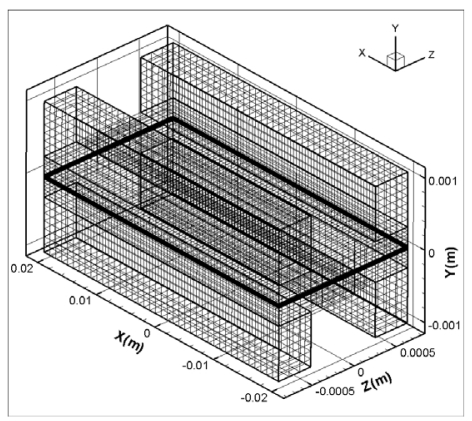
Computational grids for the micro parallel flow channels.

Figure 3 show the computational mesh structure for the micro parallel flow channel. Orthogonal non-uniform grids are employed for computational the domain. The micro parallel flow channels of anode and cathode are divided into 40 × 50 × 24 (total mesh number is 48,000). The numerical results using a mesh size twice as large as the present mesh size were almost same, so we used the present mesh size to save calculation run time.

At the inlets, the fluid is supposed to display laminar flow into the micro parallel flow channel at known velocity because Re # at anode and cathode is 900 under 100% relative humidity conditions and the atmospheric pressure is set at the outlets. Table 1 provides the physical parameters necessary for numerical calculation.

## 3. Discussions

**Figure 4.**
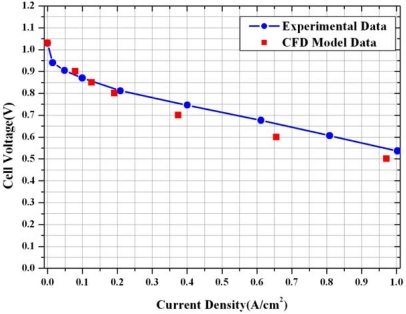
Comparison of experimental and computed polarization curves.

For a validation check of the numerical simulation model used in this study, the performance data were compared with the experimental data of a fuel cell with a parallel flow channel obtained under the same conditions as shown in Figure 4. The computed polarization curve is in favorable agreement with the experimental polarization curve [14,15].

**Figure 5.**
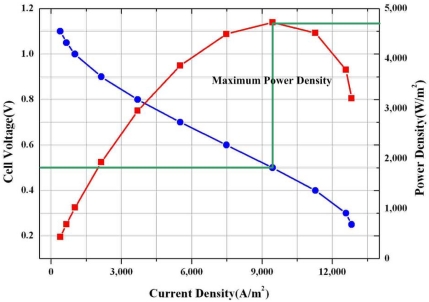
Current density(i)–Voltage(V) and power density curves.

Figure 5 presents the current density(i)–voltage(V) polarization curve for PEM fuel cell with micro parallel flow channels under conditions of 100% anode side relative humidity and 0% cathode side relative humidity. The current density increased with decreasing cell voltage. The voltage for the maximum power density at 0.5 V was found to be the optimal operation condition for the PEM fuel cell [14,15].

**Figure 6.**
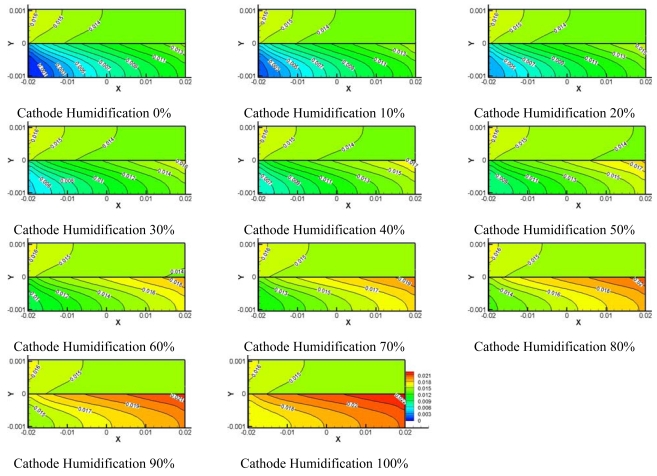
Model water concentration on center of flow channel at different cathode relative humidities.

Using this numerical simulation model and the conditions listed in Table 1, calculations were carried out to examine performance of fuel cell at operating cell voltage 0.5 V. Figure 6 shows the water concentration at the centerline surface of the channel at 0.5 V and under conditions of 0–100% cathode side relative humidity. The water concentration at the anode side shows almost the same contour, regardless of the value of relative humidity at the cathode, because 100% humidified hydrogen was supplied to the anode side. At the cathode side, the water concentration increased along the flow direction because water was generated from the electrochemical reaction and water was transported by electro-osmotic drag as oxygen flowed along the axis. The water concentration became the highest at the inlet of the flow channel when 100% cathode humidity was supplied because water concentration tends to increase at the inlet of the flow channel as the relative humidity of cathode increases.

**Figure 7.**
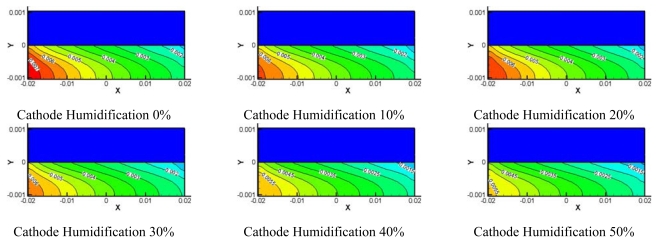

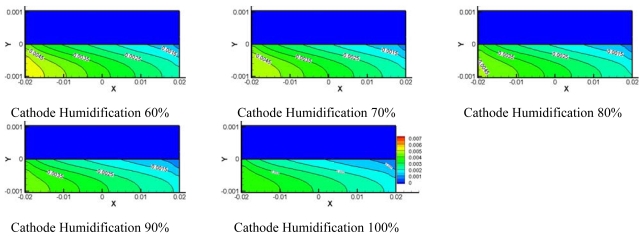
Model oxygen concentration on center of flow channel at different cathode relative humidities.

The oxygen concentration at cathode flow channel is a very important parameter to determine better performance of fuel cell. Figure 7 depicts the distribution of oxygen concentration along the centerline surface of channel. A high distribution of oxygen concentration was found at the inlet of the flow channel when the humidity of oxygen at the cathode side was low. The oxygen concentration at the cathode decreased along the flow direction regardless of humidification level at the cathode side. This decreasing distribution of oxygen concentration is thought to result from continuous water formation by oxygen and hydrogen consumption by electrochemical reaction.

**Figure 8.**
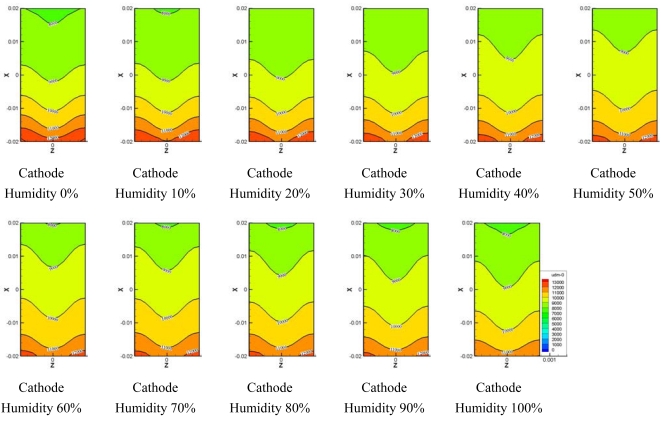
Model water concentration on anode side GDL at different cathode relative humidities.

**Figure 9.**
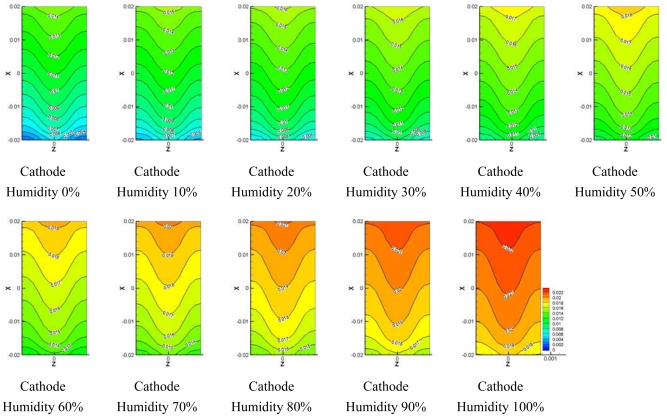
Model water concentration on cathode side GDL at different cathode relative humidities.

**Figure 10.**
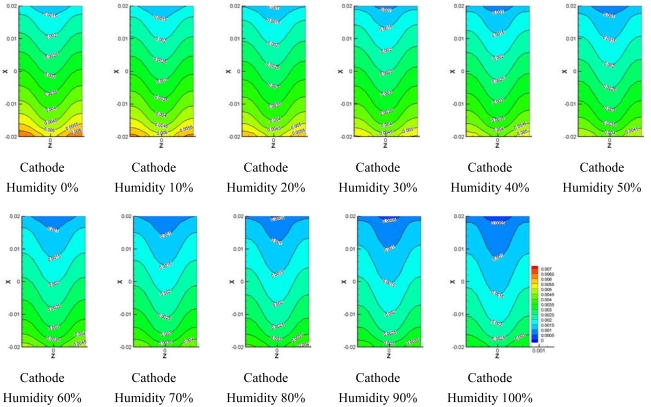
Model oxygen concentration on cathode side GDL at different cathode relative humidities.

Figures 8–10 show comparisons of the distribution of oxygen and water concentration on the gas diffusion layer at different humidity levels at the cathode channel. Figure 8 shows the distribution of water concentration on the gas diffusion layer at the anode side. Figure 9 and 10 show the distribution of water and oxygen concentration on the gas diffusion layer at the cathode side.

From comparisons for the distribution of water concentration in the gas diffusion layer at the anode side shown in Figure 8, the water concentration remains high at the flow channel inlet. The change in water concentration at the inlet and outlet of the flow channel with 0–30% of relative humidity at cathode was greater than that with 40%–100% of relative humidity at the cathode side. In addition, the distribution of water was constant, except for the inlet zone of the micro parallel flow channel in the gas diffusion layer at the anode side for case of 60%–100% of relative humidity at the cathode side.

Figure 9 shows comparisons of the distribution of water concentration in the gas diffusion layer at the cathode side. The distribution of water concentration also increased together with an increasing region of relative humidity at the cathode side. Water concentration at outlet of the flow channel increased more than water concentration at the inlet.

Figure 10 presents the comparisons of the distribution of oxygen concentration in the gas diffusion layer at the cathode side. The distribution of water concentration was opposite to the distribution of water concentration shown in Figure 10. As the humidity was increased and water concentration became greater, the proportion of oxygen and nitrogen decreased since the mixture ratio of water, oxygen and nitrogen is provided at a fixed value at the inlet of the flow channel.

**Figure 11.**
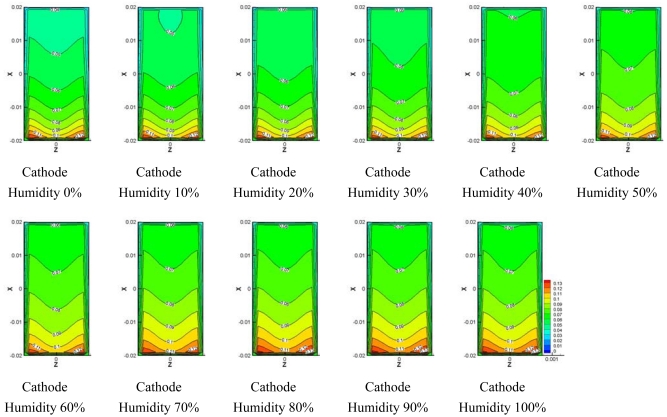
Electro-osmotic drag on the membrane at different cathode relative humidities.

**Figure 12.**
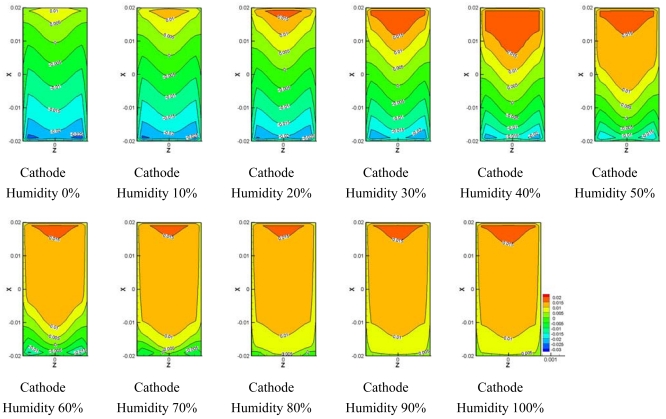
Back diffusion drag force.

Figures 11 and 12 show the water transport from the electro-osmotic drag flux and back diffusion flux. It is known that the electro-osmotic drag, which transports water molecules through the polymer membrane with hydrogen ions, moves the water molecule from the anode side to the cathode side. Water formation at the cathode resulted in a gradient in the water content between the cathode side and anode side of the membrane. This gradient caused a water flux back to anode side which was superimposed to the electro-osmotic flux.

Figure 11 shows comparisons of the electro-osmotic drag flux at different humidity levels at the cathode side. A high region of electro-osmotic drag flux was distributed at the inlet of the micro parallel flow channel, which gradually decreased along to the outlet of the flow channel. The higher relative humidity at the anode side was, the higher the electro-osmotic drag flux became at the inlet of micro parallel flow channel. Also, electro-osmotic drag flux is generated much in 50–100% of relative humidity at the cathode side at the outlet of flow channel.

Figure 12 presents the back diffusion flux at various humidity levels at the cathode side and shows that the back diffusion flux at the inlet of the flow channel was low compared to the electro-osmotic drag flux. The back diffusion flux greatly increased along to the outlet of the flow channel. The back diffusion flux remained almost constant in the region except for the inlet in the flow channel for 60%–100% of relative humidity at the cathode side.

**Figure 13.**
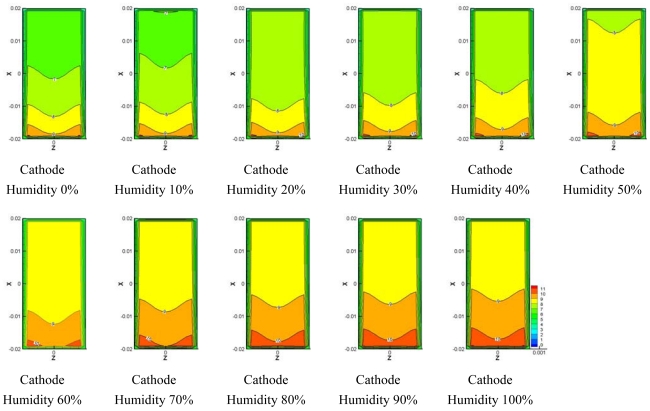
Membrane ion conductivity at different cathode relative humidities.

Figure 13 shows the ion conductivity in the membrane from variations in the different relative humidity at the cathode side. The ion conductivity of the membrane increased as the relative humidity at the cathode side increased. A region of high ion conductivity is formed at the inlet of the micro parallel flow channel, where the electro-osmotic drag flux is high. The lowest ion conductivity in the membrane was shown for the case of 0% of relative humidity at the cathode side, whereas the highest ion conduction was found in case of 100% of relative humidity at the cathode side.

**Figure 14.**
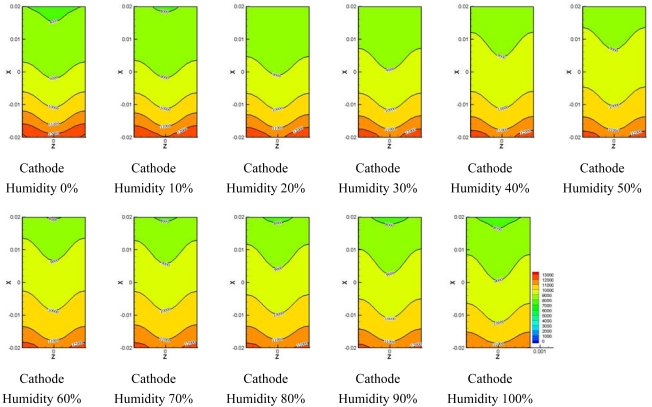
Local current density at different cathode relative humidities.

Figure 14 depicts the local current density at different relative humidity levels at the cathode side. The local current density was relatively high at the inlet of flow channel and low at the outlet of flow channel under conditions of 0–30% of low humidity at the cathode side. The entire area of the reaction had the constant level of local current density for the case of 40%–70% of humidity at the cathode side. However, the local current density was shown to be low for the case of 80%–100% relative humidity at the cathode side.

**Figure 15.**
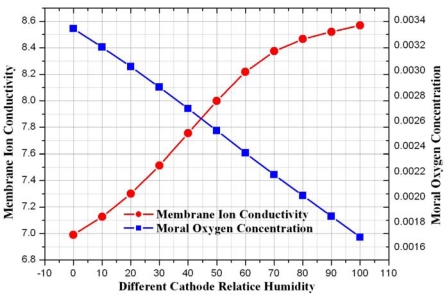
Comparison of ion conductivity and moral oxygen concentration at different cathode relative humidities.

Figure 15 presents ion conductivity and model oxygen concentration at different cathode relative humidity cathode. It is confirmed that membrane ion conductivity was increased with increasing relative humidity at the cathode relative humidity and model oxygen concentration at the cathode is found to be increased with decreasing relative humidity.

**Figure 16.**
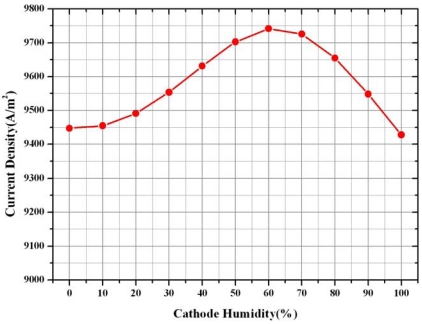
Average current density at different cathode relative humidities.

Figure 16 shows the average current density at the different relative humidity levels at the cathode side. The highest average current density is noticeable at 100% humidity at the anode side and at 60% humidity at the cathode side. Although the hydrogen ions can smoothly move from the anode side to the cathode side due to high ion conductivity in the membrane from 100% humidity at anode and cathode relative humidity, the performance of the fuel cell was not expected to be best because of the increased water concentration at cathode side resulting in the decreased oxygen concentration at cathode side. Therefore, to make a fuel cell with the best performance, there is trade-off between performance gain by increase of high ion conductivity due to high humidity and performance loss by reduction of oxygen due to high water concentration by electro osmotic drag and back diffusion in order to make best performance of fuel cell.

## 4. Conclusions

By using a fully three dimensional simulation model for a PEM fuel cell that can deal with anode and cathode flow together, the following conclusions could be obtained for a PEM fuel cell with micro parallel channelsa:

1. The computed polarization curve is in good agreement with the experimental polarization curve at low and moderate current density. At high current density, the effects of two phase flow should be considered.
2. The oxygen and water concentration on the centerline surface of the micro parallel channel and gas diffusion layer under different relative humidity at cathode side was changed greatly due to electrochemical reaction and electro osmotic drag and back diffusion.
3. The highest average current density was noticeable at 100% humidity at the anode side and at 60% humidity at the cathode side. Although the hydrogen ion can smoothly move from the anode side to the cathode side due to high ion conductivity in the membrane from 100% humidity at anode and the cathode relative humidity, the performance of fuel cell was not expected to be best because of the increased water concentration at cathode side resulting to the decreased oxygen concentration at cathode side.
4. It is found that there is trade-off between performance gain by increase of high ion conductivity due to high humidity and performance loss by reduction of oxygen due to high water concentration by electro osmotic drag and back diffusion in order to make best performance of fuel cell.

## Figures and Tables

**Table 1.** Physical parameters.

**Description**	**Value**	**Description**	**Value**
Channel length (*mm*) (*mm*)	40	Anode, Cathode side pressure (*atm*)	1
Channel width (*μm*)	762	Cell temperature (*K*)	353.15
Channel height (*μm*)	762	Anode stoichiometric number	1.5
GDL thickness (*μm*)	254	Cathode stoichiometric number	2.0
GDL porosity	0.7	*O*_2_/*N*_2_ ratio	0.21/0.79
Wet Membrane thickness (*μm*)	230	Anode side Humidification (%)	100
Catalyst layer thickness (*μm*)	28.7	Cathode side Humidification (%)	0-100

**Experimental MEA Parameters**			

Membrane	Nafion 117	Pt catalyst loading (*mg/cm*^2^)	0.4
Membrane porosity	0.28		
